# Progress and applications of single-cell RNA sequencing and spatial transcriptome technology in acute kidney injury research

**DOI:** 10.1016/j.omtn.2025.102583

**Published:** 2025-05-30

**Authors:** Chunyan Cui, Feifei Cui, Quan Zou, Zilong Zhang, Linpei Jia

**Affiliations:** 1 School of Computer Science and Technology, Hainan University, Haikou 570228, China; 2 The Affiliated Traditional Chinese Medicine Hospital, Southwest Medical University, Luzhou 646000, China; 3 Institute of Fundamental and Frontier Sciences, University of Electronic Science and Technology of China, Chengdu 610054, China; 4 Department of Nephrology, Xuanwu Hospital, Capital Medical University, No. 45 Changchun Street, Beijing 100053, China

**Keywords:** MT: Oligonucleotides: Bioinformatics, bulk RNA sequencing, single-cell RNA sequencing, spatial transcriptome technology, acute kidney injury, biomarker

## Abstract

Acute kidney injury (AKI) is a critical syndrome characterized by rapid decline in renal function. During the previous ten years, single-cell RNA sequencing (scRNA-seq) technology has evolved from a groundbreaking method to a fundamental tool in life science research. By integrating data from cellular spatial positioning with molecular omics, single-cell spatial omics offers unparalleled insight into how heterogeneous cells assemble and interact, illuminating complex networks governing cellular behavior in tissues and organs and uncovering dynamic processes of cell-cell communication. Single-cell sequencing and spatial transcriptomics have not only facilitated investigations of fundamental biological questions such as developmental differentiation but also enabled exploration of clinical issues such as cardiovascular diseases and tumor microenvironments, fundamentally transforming biomedical research paradigms. This review outlines the progression of sequencing methods, with emphasis on bulk RNA-seq, scRNA-seq, and spatially resolved transcriptomics. Additionally, we review relevant published studies to discuss the ongoing cutting-edge applications of single-cell transcriptomic profiling coupled with spatial omics in AKI research, focusing on cellular heterogeneity, personalized treatment, and other related research directions. Finally, we discuss current constraints of scRNA-seq coupled with spatial omics in AKI research. From a multi-dimensional analysis perspective, we studied scRNA-seq and spatial transcriptomics, highlighting their critical role in advancing AKI understanding.

## Graphical abstract

**Figure:**
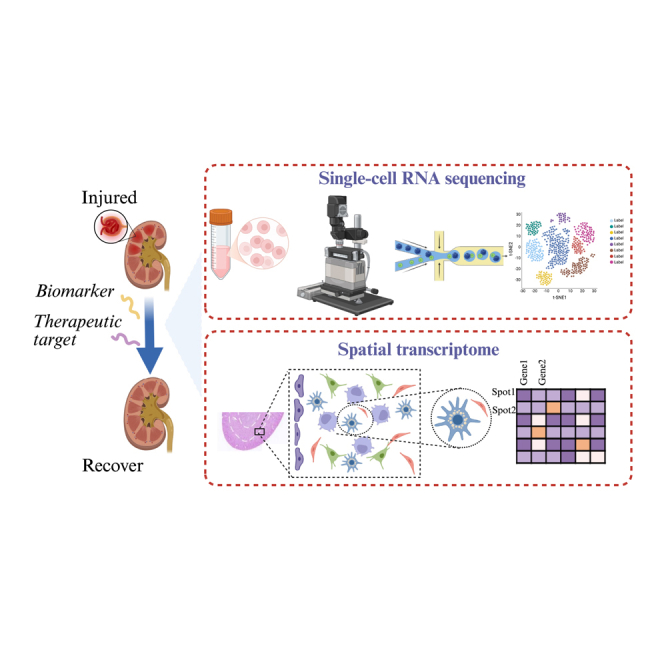

## Teaser

The development of sequencing technology, the current application status, and future challenges of single-cell sequencing and spatial transcriptome in the search for biomarkers and therapeutic targets in acute kidney injury.

## Introduction

Conventional bulk RNA sequencing (RNA-seq) usually requires extracting a sufficient amount of RNA from a tissue sample for sequencing.^1^ However, this batch RNA-seq method produces an average gene expression profile that is representative of the entire cell population. It cannot capture changes in genes within individual cells, so it is difficult to understand cellular diversity and functional changes.

With the continuous progress of technology, the sequencing resolution has been significantly improved. Unlike conventional bulk RNA-seq, single-cell RNA sequencing (scRNA-seq) captures the genetic changes in individual cells. This advanced approach facilitates detection of uncommon cellular subtypes, reveals novel cellular phenotypes, and deciphers intricate transcriptional patterns within diverse cell populations. The emergence of scRNA-seq has transformed transcriptomic studies by offering precise, high-resolution insights into tissue and organ biology.^2^ The advancement of scRNA-seq technology has profoundly transformed transcriptomics research. By facilitating a more precise and holistic analysis, it allows scientists to unravel the intricate biological properties of tissues and organs with greater accuracy and depth.^3^ By isolating individual cells and capturing transcriptomic data, scRNA-seq provides granular insights into cellular variability, elucidating nuanced variations in gene activity and functional states across distinct cell populations.^4^^,^^5^^,^^6^^,^^7^^,^^8^^,^^9^ It was first introduced by Tang et al. in 2009^10^; scRNA-seq initially analyzed the transcriptomes of 10–100 cells.^11^^,^^12^^,^^13^ As technology continues to advance, scRNA-seq technology is also continuously optimized, resulting in a range of new technologies. These innovative techniques allow researchers to simultaneously facilitate the simultaneous interrogation of transcriptomes from thousands of individual cells within a single experimental setup, greatly improving the coverage and resolution of sequencing.^14^^,^^15^ In 2013, *Nature Methods* named single-cell genomics and transcriptomics “Methods of the Year,” predicting that these breakthrough technologies will drive profound advancements in our comprehension of cellular heterogeneity. Five years later, single-cell sequencing enabled time tracking of more than 20 cell types in zebrafish embryos, which was named the top achievement of the year by *Science*. In the following two years, *Nature Methods* named single-cell multiomics and spatial transcriptomics “Methods of the Year,” further cementing the critical role of single-cell sequencing in advancing life science research.

Since its initial use for genome and transcriptome analysis, scRNA-seq has evolved rapidly, enabling profound investigations across diverse research domains, including epigenomics, three-dimensional genomics, and spatial transcriptomics. In 2016, Ståhl et al.^16^ introduced the groundbreaking technology of spatial transcriptomics, which has rapidly appeared as a breakthrough technology since its inception. Spatial transcriptomics enables the acquisition of transcriptomic data from whole tissue slices while providing spatial localization information, thereby addressing the limitations of scRNA-seq that lacks spatial resolution.^17^ Currently, spatial transcriptomic techniques currently serve as valuable tools for examining assorted tissue varieties, particularly in studies investigating tumor heterogeneity.^18^^,^^19^^,^^20^^,^^21^ Furthermore, spatial transcriptomics has facilitated breakthroughs in diverse research fields and perspectives, encompassing cell clustering and intercellular communication, metabolic dynamics, transcriptional activity, immunological cell reprogramming, and cross-modal technology integration, for example, spatial proteomics, spatial epigenomics, etc.^22^^,^^23^^,^^24^^,^^25^^,^^26^ The rapid accumulation of large-scale single-cell data has provided unprecedented resources for extracting biological information from experimental data, ultimately leading to the generation of novel biological insights, thus solidifying the data foundation for new discoveries in life sciences. The analysis of these data, propelled by significant advancements in information technologies such as deep learning, has yielded fruitful results. Various clustering algorithms have been employed to identify cell types with similar characteristics, revealing the cellular composition within organs.^27^^,^^28^^,^^29^ This hierarchical representation of cell types at the systems, organ, and tissue levels has formed a cellular atlas that serves as a microcosm of life. Notably, the Human Cell Atlas initiative, which seeks to characterize the approximately 50 trillion cells in the human organ systems, has garnered a positive response from the global scientific community since its proposal in 2017.

Acute kidney injury (AKI) is characterized by an acute impairment of kidney function that worsens within hours to days, representing a critical medical condition with multifactorial etiology, which strongly correlates not only with a significant increase in mortality and hospitalization rates but also with the potential to trigger or exacerbate chronic kidney disease (CKD), ultimately progressing to end-stage kidney disease.^30^^,^^31^ Elucidating the molecular pathogenesis of AKI during its initial phases is indispensable for developing targeted therapeutic strategies. The pathophysiology of AKI involves coordinated interactions among multiple renal cellular components, notably tubular epithelial cells, glomerular podocytes, vascular endothelial cells, immune effector cells (e.g., macrophages and neutrophils), and interstitial stromal cells. Each cellular population exhibits distinct pathological contributions to disease initiation and progression.^32^ scRNA-seq and spatial transcriptomics provide cutting-edge techniques for studying cell complexity in AKI. Through these advanced techniques, researchers are able to deeply analyze the different states of individually profiled cells and the heterogeneity between cells, thereby revealing more fully the biological processes and molecular mechanisms associated with AKI occurrence. In this study, we present a thorough overview of several major sequencing techniques currently used in AKI research and discuss in detail strengths and constraints of the respective techniques. In addition, this study also discusses some of the current challenges in the practical application of these technologies, especially in the aspects of data analysis, standardization of technology platforms, and clinical translation. In order to make these technologies play a greater role in AKI research and clinical treatment, overcoming these technical difficulties emerges as a high-priority area of inquiry for future studies.

## Development of sequencing technology

**Figure 1:**
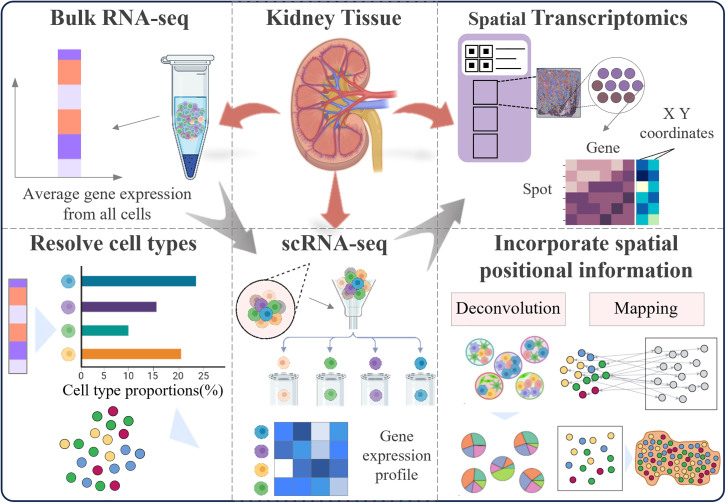
Development of sequencing technology As technology continues to advance, a variety of sequencing techniques can now be applied to kidney tissue sections, such as bulk RNA sequencing, single-cell sequencing, and spatial transcriptomics. Bulk RNA sequencing provides overall gene expression levels but averages signals across cells, failing to capture cellular heterogeneity. Single-cell sequencing addresses this challenge by examining gene expression at the level of individual cells, uncovering distinct cell types and their respective functions. However, it loses spatial context. Spatial transcriptomics overcomes this by associating gene expression with its spatial context, allowing the investigation of gene distribution across tissues while maintaining their structural integrity. Moreover, single-cell sequencing data enables detailed analysis of heterogeneous cell populations within bulk samples. Through deconvolution algorithms and mapping algorithms for spatial localization, spatial transcriptomic data can help to accurately infer the cell composition within each spatial location or tissue region, and even further analyze how these cells interact spatially.

In the 1980s, the traditional RNA-seq technology mainly relied on the Sanger sequencing method, and usually only specific RNA molecules, especially messenger RNA (mRNA), were used as the object of study. During this period, research was mainly focused on the quantitative and qualitative evaluation of gene expression, such as the identification of differentially expressed genes (e.g., development or disease-related genes), splice variant analysis (through cDNA library screening), and sequencing of viral RNA genomes (such as influenza virus and HIV).^33^^,^^34^^,^^35^ However, Sanger sequencing is unable to comprehensively capture low-abundance transcripts and is difficult to reveal the heterogeneity among cells. The landscape of genomic research underwent a dramatic evolution in 2005, coinciding with the debut of high-throughput sequencing systems by companies such as Illumina and 454 Life Sciences, which significantly enhanced the efficiency of RNA sequencing.^36^^,^^37^ RNA-seq technology was introduced, achieving digital quantification of gene expression. It can sequence all transcripts in the sample, no longer limited to specific genes, and is capable of identifying low-abundance transcripts and rare splicing variants. At this stage, research began to focus on the dynamic changes in transcriptomics (such as time series analysis) and the functions of non-coding RNAs, laying the foundation for subsequent single-cell technologies. In 2009, Tang et al. pioneered scRNA-seq using droplet technology, marking a significant milestone in the area of single-cell analysis.^10^ This technology can reveal cellular heterogeneity, analyze the differences in gene expression among different cells, resolve developmental trajectories, and reconstruct the cell differentiation process through pseudo-temporal analysis (such as Monocle). Subsequent technologies (such as 10× Genomics) have further enhanced throughput and cost efficiency, making scRNA-seq become a core tool for studying complex tissues, such as kidneys and brains. In 2016, Ståhl et al. presented a method that combined transcriptome sequencing with tissue sectioning, enabling the capture of RNA spatial distribution within tissues.^16^ Subsequently, technologies such as Space-seq and Visium were developed, integrating traditional transcriptomic sequencing with spatial localization approaches.^38^ By preserving spatial coordinates of RNA molecules within intact tissue architectures, spatial transcriptomics enables systematic investigation of gene activity patterns across distinct histological regions. This approach overcomes the spatial resolution limitations inherent to conventional bulk RNA-seq and scRNA-seq methods, thereby establishing critical linkages between molecular profiles and tissue microanatomy. It analyzes the organizational microenvironment by retaining spatial location information and can achieve multimodal integration by combining histomorphology, spatial epigenomics, spatial genomics, and spatial proteomics. For a specific illustration, refer to Figure 1.

## Applications in AKI research

The use of scRNA-seq technology to study AKI has attracted much attention from researchers and clinicians. The scRNA-seq facilitates in-depth transcriptome analysis of distinct renal cell subtypes at the single-cell scale, thus providing more accurate sequencing results.^39^^,^^40^^,^^41^ The pathophysiological mechanism of AKI is extremely complex, involving multiple cell types and their interactions. Traditional batch tissue sequencing methods, which typically analyze the entire kidney tissue as a whole, can provide a holistic picture of gene expression, but often fail to account for the intrarenal cellular diversity and cell-type-specific pathophysiological mechanisms driving AKI progression. In contrast, the scRNA-seq technique provides unprecedented technical support for studying transcriptomic profiles of individual cells within renal tissues.^42^^,^^43^ This unique ability of scRNA-seq enables researchers to systematically explore the distinct functions and roles of specific cell types in AKI. In addition, scRNA-seq can not only reveal transcriptional changes inside cells but also help us understand signaling and interactions between different cells.^44^^,^^45^^,^^46^ The employment of this technology in clinical diseases offers foundational insights for developing temporally sensitive detection methods and patient-specific intervention protocols in AKI management, improves clinical management, and improves the treatment effect and overall quality of life in patients.

**Figure 2:**
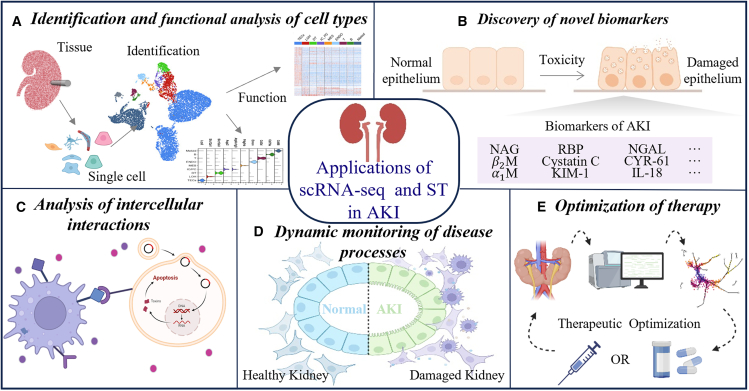
Single-cell RNA sequencing and spatial transcriptomics in acute kidney injury research (A) Identification and functional analysis of cell types: using scRNA-seq transcriptomic techniques, the different cell types in the kidney and their distribution in specific spatial regions are precisely identified. (B) Discovery of novel biomarkers: by comparing gene expression profiles in healthy and injured tissues, potential novel biomarkers associated with acute kidney injury were identified. (C) Analysis of intercellular interactions: revealing the signaling pathways and interaction mechanisms between different cell types in the kidney. (D) Dynamic monitoring of disease processes: tracking and monitoring the progression of acute kidney injury by comparing healthy and damaged kidney tissue in real time, capturing critical moments in the development of the disease. (E) Optimization of therapy: according to the analysis results, the treatment plan is adjusted and optimized.

The adoption of scRNA-seq in AKI investigations has increased exponentially, because it can deeply reveal the heterogeneity and different functional states and cell-cell interactions of kidney cells at the single-cell precision, in order to provide a more thorough and detailed theoretical basis for in-depth understanding of the pathophysiological process of AKI. On this basis, the spatial transcriptome has more advantages. First, spatial transcriptomics offers a unique advantage over scRNA-seq in that it not only can enables simultaneous transcriptional profiling of individual cells, but also preserve their native spatial coordinates within intact tissue architectures, further enhancing the understanding of tissue structure and cell interactions.^47^^,^^48^ Second, this technique can more deeply reveal the influence of the local microenvironment to capture the dynamic changes in cell types during AKI.^48^^,^^49^ In addition, spatial transcriptomics technology can effectively identify cell heterogeneity within the tissue slice by direct analysis of the *in-situ* tissue slice of clinical samples, and avoid the sample processing errors that may occur in traditional methods.^50^^,^^51^ In the study of AKI, the application of scRNA-seq technology and spatial transcriptomics mainly focuses on the following key areas: (1) identification and functional analysis of cell types; (2) discovery of novel biomarkers; (3) analysis of intercellular interactions; (4) dynamic monitoring of disease processes; and (5) optimization of therapy. For a specific illustration, refer to Figure 2.

## Identification and functional analysis of cell types

Advances in single-cell transcriptomic profiling have empowered investigators to delineate renal cellular subpopulations with unprecedented resolution, elucidating their functional contributions to AKI pathogenesis. For example, Yao et al. used scRNA-seq to resolve the diversity of macrophage phenotypes in models of ischemia-reperfusion injury (IRI-AKI), especially significant differences in the subclass and functional status of macrophages compared to mice undergoing sham surgery. The results suggest that S100a9^hi^Ly6c^hi^ monocytes serve a critical function in the initiation and development of renal inflammatory cascades in the early stage of AKI. In addition, the study further validated the strong correlation between S100A8/A9^+^ macrophages in renal infiltration and tissue damage, indicating the critical role of these cellular participants in inflammatory cascades. The study also revealed the important role of renal resident macrophages (KRM) and infiltrating macrophages derived from monocytes in AKI.^52^ In a pivotal study, Chen et al. performed scRNA-seq on murine renal tissues following induction of five major AKI etiologies: cisplatin nephrotoxicity (CP), ischemic reperfusion injury (IRI), unilateral ureteral obstruction (UUO), folic acid toxicity, and sodium oxalate (SO) crystals. Their transcriptomic analysis demonstrated that maladapted proximal tubule cells (PTCs) and classical Havcr1^+^PTCs, not the recently characterized Krt20^+^PTCs subset, serve as primary drivers of both inflammatory activation and fibrogenesis in multiple AKI paradigms.^53^ Hinze et al. performed high-resolution single-cell transcriptomic profiling of human AKI-affected renal tissues. Their investigation identified previously unrecognized injury-responsive cellular phenotypes across principal tubular epithelial populations, with most prominent transcriptional alterations observed in proximal tubules, thick ascending limbs, and distal convoluted tubules. Through their detailed examination, they identified four distinct injury-related cellular states, each exhibiting unique transcriptomic signatures. These states were hierarchically interconnected and characterized by specific molecular pathways. Notably, the transcriptomic profiles were linked to key pathogenic processes, including oxidative stress, low-oxygen adaptation, the interferon response, and epithelial-to-mesenchymal transition (EMT).^54^ Do Valle Duraes et al. performed an unbiased scRNA-seq analysis to investigate immune cell changes following AKI. Significant changes were observed in tissue-resident macrophages and T cells, particularly with an increased presence of IL-33R^+^and IL-2Ra^+^regulatory T cells (Tregs).^55^ Creed et al. used scRNA-seq technology to determine the behavior of renal lymphatic endothelial cells in response to AKI.^56^ Rao et al. identified a previously unrecognized macrophage subpopulation exhibiting transitional phenotypes, which was specifically associated with the pathogenesis of rhabdomyolysis-mediated AKI, distinguished by the activation of cellular aging processes, through scRNA-seq.^57^ Li et al. induced an ischemia-reperfusion AKI model in VE-PTPKO mice. Single-cell transcriptomic profiling identified distinct glomerular endothelial cell clusters that were uniquely present in AKI models but absent in sham-operated controls, suggesting injury-specific vascular remodeling.^58^ Wen et al. performed scRNA-seq on renal allograft biopsies, capturing 81,139 individual cells from three antibody-mediated rejection and two rejection-associated AKI patients, establishing a comprehensive cellular atlas of transplant injury. They identified 11 cell categories and characterized the subpopulations of each cell type, revealing the immune profile of AKI caused by kidney transplant rejection.^59^ Saxena et al. employed scRNA-seq to identify kidney intercalated cells as a renal epithelial subpopulation demonstrating significant transcriptional remodeling during AKI, suggesting their functional involvement in disease pathogenesis.^60^ Cheung et al. employed a comprehensive multi-omics strategy, incorporating single-cell transcriptomics, high-dimensional flow cytometry, spatially resolved gene expression profiling, and multiplexed immunofluorescence to identify, describe, and confirm kidney-resident macrophages (KRMs) populations at rest and 19 min post bilateral ischemic kidney damage. They revealed seven distinct KRM subpopulations organized in correspondence with renal unit regions leveraging scRNA-seq alongside spatial transcriptomics techniques.^48^ Utilizing high-dimensional transcriptomic profiling, Gharaie et al. systematically characterized renal TCR^+^CD4^−^CD8^−^ (double-negative [DN]) T cells populations in murine models of IRI-AKI. Their comparative analysis revealed that unlike conventional CD4^+^and CD8^+^ T cells in homeostatic kidneys, injury-responsive DN T cells acquired distinct immunoregulatory signatures. They comprehensively characterized the transcriptional signatures and tissue distribution patterns of DN T cells, revealing their unique immunoregulatory properties.^46^ Zhang et al. downloaded scRNA-seq data of acutely damaged or normal kidneys from the GEO database, identifying resident progenitors marked by Top2a that are controlling PT function restoration of ischemia-reperfusion-induced AKI.^61^

## Discovery of novel biomarkers

Using scRNA-seq technology, researchers have been able to identify novel biomarkers with high potential for early detection and prediction of the occurrence and progression of AKI. By conducting in-depth analyses of the cellular transcriptomes before and after kidney injury, researchers can determine which genes are significantly upregulated or downregulated in the early stages of damage. These gene expression changes not only reveal the immediate responses of renal cells to injury but also provide critical clues for discovering potential diagnostic aids and treatment targets. As a case in point, Tang et al. utilized scRNA-seq analysis in the kidneys to characterize the transcriptomic landscape of AKI patients, discovering previously unreported pro-apoptotic genes in the injured PT cells, which unveil the cell-specific gene expression profile of the kidneys in ischemic AKI and provide new insights into potential therapeutic targets.^62^ Li et al. adopted an integrative multi-omics strategy, merging simultaneous high-throughput single-cell ATAC/RNA sequencing with spatially resolved metabolic profiling. This approach was applied to analyze 54 human kidney specimens across diverse anatomical regions, integrating multifaceted omics datasets with clinical metadata. Through this comprehensive analysis, the team identified PLEKHA1 as a potential disease biomarker.^63^ Wang et al. used scRNA-seq analysis to characterize macrophage subtypes related to ischemia-reperfusion-induced AKI and then filtered relevant key genes using cross-sectional bulk RNA-seq data, finding that Ier3 may play an essential role in AKI, with S100a6, Vim, Ier3, and Ccr1 potentially serving as new biomarkers and treatment target for ischemia-reperfusion renal injury (IRI)-induced AKI.^64^ Li et al. investigated scRNA-seq data in rodents models of AKI induced by UUO, ischemia-reperfusion (IR), cisplatin (CP), SO, and lipopolysaccharide, discovering that Gclc may serve as a potential biomarker for distal nephron damage.^65^ Through publicly available scRNA-seq datasets, Peng et al. identified macrophage-specific Saa3 as a key mediator of inflammatory responses in sepsis-associated AKI, suggesting its potential utility as a diagnostic biomarker.^66^ Wang et al. searched the GEO database for AKI single-cell sequencing datasets, determining that SLC2A1 is a diagnostic biomarker for AKI.^67^

## Analysis of intercellular interactions

Single-cell sequencing technology serves as a powerful means for uncovering underlying biological mechanisms of intercellular communication and signaling. Researchers can delve into the complex interactions between diverse cell types during the procedure of AKI through paired analyses between cells. By identifying and analyzing intercellular ligand-receptor pairs, investigators can infer how tubular cells and immune cells mutually regulate each other through molecular signals. These signaling mechanisms may lead to either exacerbation or alleviation of inflammation, thereby affecting the progression of injury. For example, after AKI, patients either heal or advance to fibrosis and CKD. Kirita et al. utilized scRNA-seq platforms to detect a condition of impaired repair in proximal tubular cells (FR-PTC) and described a dynamic network of intercellular communication, also providing a comprehensive overview of cellular reactions after injury.^68^ Although single-cell transcriptomics has elucidated cell-type-specific molecular profiles in renal tissues, the spatial organization of AKI pathology and its cell-type-dependent effects exhibit significant regional variation across nephron segments. Dixon et al. employed a combined approach of scRNA-seq and spatial transcriptomic profiling discovering that even six weeks post-injury, injured proximal tubular cells showed enhanced interactions with macrophages and lymphocytes. They delineated topographically distinct injury signatures, observed the disappearance of damage-responsive differentiation markers, and documented their subsequent re-emergence during renal regeneration.^69^ Zhang et al. used an unbiased scRNA-seq system to explore AKI caused by ischemia-reperfusion, uncovering mechanisms of epithelial-immune cell interactions.^70^

## Dynamic monitoring of disease processes

Single-cell sequencing can also enable real-time monitoring of the progression of AKI. In animal models or clinical samples, researchers can obtain single-cell data over time to analyze dynamic cellular changes, thereby understanding the different stages and prognosis of AKI. For instance, transitions between cell populations at the onset and the recovery phase can be observed, characterizing specific cell types and underlying signaling cascades involved in the recovery process. For example, Li et al. employed scRNA-seq technology to identify the different injury states of proximal tubules and investigated the epithelial cells of the renal unit segments to identify common and unique injury responses.^71^ Klocke et al. established a non-invasive urinary cell sorting workflow coupled with single-cell transcriptomics, wherein flow-sorted renal cells underwent scRNA-seq analysis. This innovative methodology enables longitudinal monitoring of AKI progression at cellular resolution, demonstrating clinical applicability for renal injury assessment. Single-cell transcriptomic analysis of renal cells shed in urine offers new opportunities to monitor disease progression and therapeutic interventions.^72^ Hasegawa et al. reported that urine from AKI patients contains injured renal tubular epithelial cells with dedifferentiated and adaptive phenotypes, reflecting renal tissue damage.^73^ The findings of Rudman-Melnick et al. demonstrated the temporal progression of changed patterns of gene expression across numerous phases of AKI and all varieties of renal cell types, providing valuable resources for examining disrupted molecular pathways in AKI.^74^ Shan et al. conducted scRNA-seq on a clinically relevant IRI mouse-based experimental model with varying ischemic durations, revealing dynamic cellular changes leading to AKI.^75^ Huang et al. applied scRNA-seq in combination with immunostaining to patient biopsies, showing that persistent upregulation of fibroblast activation protein in the kidneys after AKI is linked to maladaptive repair and fibrosis.^76^

## Optimization of therapy

Single-cell transcriptomics has become a foundational technology in AKI research, enabling precise molecular characterization of injury mechanisms and informing targeted therapeutic strategies. By analyzing the heterogeneity and functions of renal cells, this technology advances precision medicine, significantly improving patient outcomes. For instance, Wang et al. used scRNA-seq to demonstrate the therapeutic role of mesenchymal stem cells in AKI, helping to refine future treatment strategies.^77^ De Chiara et al. pioneered an integrative approach combining DNA quantification with scRNA-seq, mapping ploidy variations in renal tubular epithelial cells during AKI progression. Their multi-omics strategy uncovered a druggable pathway that ameliorates post-AKI renal fibrosis in preclinical models.^78^ Through single-cell transcriptomic profiling of murine and human AKI kidneys, Noel et al. discovered T cell-specific overexpression of TIGIT, an immune checkpoint regulator, positioning this pathway as a novel therapeutic target for renal injury intervention.^79^ Hong et al. discovered that WT1^+^PECs (a subtype of glomeruli parietal epithelial cells) promote the regeneration and repair of proximal tubular cells following severe AKI, highlighting a potential target for AKI repair.^80^ Through integrating single-cell transcriptome and spatial metabolomics analysis, Li et al. revealed the core role of Nrp1^+^distal renal tubular cells in AKI fibrosis. These cells drive myofibroblast activation by activating Smad3-dependent signaling pathways. Further studies have shown that targeting NRP1 provides a new target for blocking the developmental trajectory of AKI to CKD.^81^ Wang et al. conducted scRNA-seq on a unilateral ischemia-reperfusion (UIR) murine AKI models at post-injury time points (1, 3, 14, and 28 days), suggesting that targeting the Mincle receptor on macrophages and neutrophils could effectively prevent the developmental trajectory from AKI to CKD.^82^ Through scRNA-seq, Song et al. demonstrated that pharmacological neutralization of PSMP markedly attenuated renal damage *in vivo*, establishing this pathway as a promising therapeutic target for AKI.^83^ Zhang et al. joined scRNA-seq and spatially resolved transcriptomics to reveal the spatiotemporal heterogeneity of macrophages during the AKI-to-CKD transition, identifying extracellular matrix remodeling-associated macrophage subsets as promising therapeutic targets for CKD prevention.^84^ Yu et al. employed an integrative multi-omics approach combining whole-genome bisulfite sequencing, MeRIP-seq (m6A profiling), and single-cell transcriptomics to identify epigenetic dysregulation in renal S3 proximal tubules of vitamin C-deprived mice. Their findings imply that vitamin C repletion could mitigate DNA/RNA methylation-associated tubular injury.^85^ Jiang et al. employed a unilateral ischemia-reperfusion injury (uIRI) model to map T cell dynamics at single-cell resolution during AKI-CKD progression. Their findings demonstrated that selective depletion of CD8^+^ T cells attenuated peritubular capillary rarefaction and interstitial fibrosis, implicating cytotoxic T cells as key mediators of microvascular loss in chronic renal injury.^86^ Chen et al. developed an AKI model in Sox9 lineage tracing mice treated with PGE2 and performed scRNA-seq. They discovered that PGE2 activates renal Sox9^+^ cells, driving their differentiation into proximal tubular epithelial cells while inhibiting fibrosis, underscoring PGE2’s potential for AKI regenerative therapy.^87^ Chu et al. employed spatially resolved transcriptomic profiling to delineate GPX4 expression patterns at the endothelial-outer medullary junction. Their analysis revealed compartment-specific ferroptosis signaling attenuation during renal IRI-AKI, suggesting GPX4 modulation as a viable therapeutic strategy for I/R-associated nephropathies.^88^ Liu et al. used scRNA-seq to reveal the role of platelet-driven circulating macrophages in treating renal fibrosis, identifying platelet-derived thrombospondin 1 (THBS1) as an actionable target for suppressing inflammatory cascades and fibrotic in renal tissue.^89^

Single-cell sequencing and spatial transcriptomic analysis have paved the way for innovative strategies to address therapeutic challenges in AKI. At the level of cell characteristic analysis, these innovative methods have for the first instance comprehensively demonstrated the differential response characteristics of renal parenchymal cells to injury, especially the response differences of cells in different functional segments of the PT (including S1–S3 regions) under hypoxic conditions, and identified the population of SOX9-positive precursor cells with repair potential. Within the domain of diagnostic marker research and development, the application of spatial positioning technology has effectively solved the problem of unclear cell sources of traditional indicators and established a new early warning system based on the characteristics of the tissue microenvironment. Through the study of intercellular communication, the promoting effect of key regulatory networks, including macrophage-epithelial cell dialogue, on the progression of injury was clarified. Time series tracking technology has successfully captured the key transformation nodes of cell states during the disease development process, creating a valuable opportunity window for clinical intervention. Ultimately, these findings guided the design of precise treatment plans for different subtypes of AKI. These breakthroughs have jointly constructed a new research paradigm of “single-cell typing-spatiotemporal dynamic analysis-precise intervention,” fundamentally redefining the molecular and cellular heterogeneity of AKI, uncovering dynamic disease trajectories that provide a framework for precision therapeutic development.

## Discussion

Over the past decade, scRNA-seq technology has undergone significant advancements driven by multiple innovations, including more sensitive single-cell isolation methods, automation technologies, cost-effective experimental processes, and high-throughput sequencing platforms. Currently, researchers face various scRNA-seq methods with unique characteristics, allowing them to select the most suitable technology based on specific research objectives and sample features.^90^^,^^91^^,^^92^ These methods include not only classic separation and sequencing strategies but also emerging technologies such as spatial transcriptomics, enabling researchers to retain the topographic organization of cellular architectures in tissue specimens and conduct in-depth studies of the cellular microenvironment.^93^^,^^94^ By analyzing individual cells within complex cell populations, scRNA-seq demonstrates its unique advantages, particularly in revealing the heterogeneity of cell populations.^95^^,^^96^^,^^97^ Furthermore, the scRNA-seq technology is profoundly changing modern medical practice and providing unprecedented technical support for the development of personalized medicine. By elucidating the specific characteristics of cells in patient samples, doctors can tailor more precise treatment plans for patients.^98^^,^^99^^,^^100^ The ongoing advancement of scRNA-seq technology not only offers unprecedented biological insights but also opens new pathways for future clinical applications. The potential of this technology will play a pivotal part in fundamental research, pathological assessment, and therapeutic intervention driving ongoing progress in cell biology and precision medicine.

Single-cell genomics and spatial transcriptomics are advancing at a rapid pace, moving toward greater precision and higher throughput. The progress of these new technologies, especially the launch of commercially available large-scale single-cell sequencing technologies, including the 10× Chromium X platform,^101^ allows researchers to profile a greater number of cells in a single assay, detect rare cellular subsets, and substantially enhance the resolution of tissue characterization.^3^^,^^102^^,^^103^ Today, researchers can detail the gene expression signatures of different cellular categories and examine the differences in genetic expression between comparable cell types under healthy and diseased conditions.^40^^,^^104^^,^^105^ Additionally, through these technologies, scientists can infer cell differentiation trajectories, gaining deeper insights into the activity of transcription factors or signaling pathways during tissue development.^106^^,^^107^^,^^108^ Meanwhile, these analytical tools also enable us to predict interactions between cells, unveiling unprecedented insights into cell communication and its multifaceted roles in maintaining health and triggering disease.^109^^,^^110^ In summary, advancements in single-cell genomics and spatial transcriptomics methodologies have substantially improved our comprehension of cellular processes. These technological breakthroughs not only deepen our knowledge base in cell biology but also serve as a foundation for the progress of individualized medical approaches. With the potential to revolutionize the field, they may well mark the beginning of a transformative phase in biomedical research.

Examining normal renal tissue at the single-cell resolution offers critical insights into the developmental processes of the kidney and the functional mechanisms that underpin its biological activities.^40^^,^^52^ However, single-cell experiments often generate massive datasets that far exceed what a single research paper can handle. These data explosion phenomenon makes the storage, management, and sharing of data a significant challenge in single-cell research. Therefore, the deposition of generated data and its subsequent utilization become particularly important. To address this challenge, researchers are increasingly recognizing that reanalyzing existing datasets using advanced bioinformatics tools or integrating multiple existing datasets can significantly enhance the value of the data.^111^^,^^112^^,^^113^^,^^114^^,^^115^ For instance, combining scRNA-seq data from different studies, researchers can more comprehensively depict the developmental trajectories of kidney cells and their alterations under various physiological and pathological states.^66^^,^^67^ This approach not only provides deeper insights into renal biology but also establishes the foundation for creating innovative therapeutic approaches. Thus, data generation, storage, and integration in single-cell research are not only crucial components of scientific investigation but also key factors driving the continuous advancement of kidney research.

At present, high-throughput scRNA-seq technology and spatial transcriptomics are exerting a profound influence on nephrology. The core advantage of this technology is rooted in its capacity to thoroughly characterize every cellular type and their functional states, a feature that may fundamentally reshape our understanding of various heterogeneous diseases such as AKI, CKD, and transplant rejection.^49^^,^^59^^,^^116^ Through meticulous cellular analysis, scRNA-seq holds the potential to offer more accurate diagnostic methods, reliable prognostic biomarkers, and identify signaling pathways that could be leverage for targeted therapies. Furthermore, the construction of gene expression maps or systemic molecular classification of the kidney has emerged as a definite objective of precision medicine projects for the kidney.^117^^,^^118^ Through such precise gene expression analysis, physicians can better discern the underlying mechanisms of the disease, thereby formulating individualized treatment plans and enhancing treatment efficacy. More significantly, these methodologies are equally applicable to the research of kidney organoids, enabling pluripotent stem cells to efficiently differentiate into various renal cell types.^119^^,^^120^ With an enhanced understanding of the characteristics of kidney cells, researchers can optimize the differentiation process and enhance the functionality and physiological relevance of kidney organoids. This is not only of great significance for fundamental research but also offers new notions and possibilities for regenerative medicine in kidney diseases.

Currently, within the realm of AKI investigations, the utilization of scRNA-seq and spatial transcriptomics methodologies faces multiple challenges: In terms of sample acquisition, renal tissue biopsy of AKI patients is difficult, and ischemic injury leads to fragile cells that are prone to death during dissociation. Currently, the cell survival rate can be improved by optimizing the cold digestion method. Although cold digestion can increase cell survival rate, it can lead to the selective loss of specific segments of renal tubular epithelial cells (such as the S3 segment).^121^ Technically, renal cells are highly heterogeneous (such as different segments of renal tubules), and scRNA-seq has difficulty in completely distinguishing subtle subgroups. Moreover, the resolution of spatial transcriptome (such as 55 μm in Visium) is not sufficient to resolve the precise expression profile of a single renal tubule (with a diameter of ∼20 μm). At present, high-depth sequencing (>50,000 reads/cell) combined with CITE-seq protein labeling can be adopted to improve the clustering ability, and Xenium (with a resolution of 150 nm) or MERFISH can be used to achieve subcellular localization. However, the ultra-high resolution technolog such as Xenium (150 nm) can currently only detect 300–500 genes, missing key AKI pathway genes.^122^ In terms of data analysis, it is hard to capture the instantaneous state of the dynamic injury process, and there is a lack of a unified annotation standard for renal cells. Currently, this problem can be solved by combining time series sampling (such as 0 h/6 h/24 h) with pseudo-time analysis (Monocle3). However, what followed was that Monocle3 generated approximately 30% of false positive trajectory branches in AKI dynamic modeling.^123^ The bottleneck of clinical transformation lies in the differences between animal models and human AKI, along with the insufficiency of multi-omics data integration methods. Future research urgently needs to break through the current technical bottlenecks through multi-dimensional technological innovation: At the sample processing level, a kidney-specific mild dissection scheme should be developed, and low-damage enzyme combinations and microfluidic sorting technology should be adopted to ensure cell survival rate while preserving the integrity of key cell subsets. At the technical level, it is necessary to promote the development of ultra-high-resolution spatial multi-omics technology, integrate the *in situ* detection capabilities of single-cell transcriptomics, proteomics and metabolomics, and achieve subcellular level analysis of different segments of nephrons. At the data analysis level, it is necessary to construct a bioinformatics process specific to the kidneys, establish cross-species cell annotation standards, and develop an AI algorithm system integrating dynamic modeling and clinical prediction.

Overall, the evolution of scRNA-seq alongside spatial transcriptomic methodologies has brought revolutionary changes to nephrology. These advancements not only deepen our comprehension of intricate biological mechanisms of the kidneys but may also drive progress in precision medicine and regenerative medicine, providing new hope and direction for developing novel therapeutic strategies in kidney disease treatment.

## Acknowledgments

The authors declare that this manuscript does not involve animal or human experiments.

The work was supported by the 10.13039/501100001809National Natural Science Foundation of China (nos. 62131004 and 62402403). Graphical abstract and Figures 1 and 2 were created with BioRender (https://www.biorender.com/).

## Author contributions

C.C., writing – original draft and funding acquisition. F.C., investigation and methodology. Q.Z., funding acquisition and writing – review & editing. Z.Z., writing – review & editing and supervision. L.J., writing – review & editing and supervision.

## Declaration of interests

The authors declare no competing interests.
